# Effects of Surface Asymmetry on Neuronal Growth

**DOI:** 10.1371/journal.pone.0106709

**Published:** 2014-09-03

**Authors:** Elise Spedden, Matthew R. Wiens, Melik C. Demirel, Cristian Staii

**Affiliations:** 1 Department of Physics and Astronomy and Center for Nanoscopic Physics, Tufts University, Medford, Massachusetts, United States of America; 2 Materials Research Institute and Department of Engineering Science, Pennsylvania State University, University Park, Pennsylvania, United States of America; Institut de la Vision, France

## Abstract

Detailed knowledge of how the surface physical properties, such as mechanics, topography and texture influence axonal outgrowth and guidance is essential for understanding the processes that control neuron development, the formation of functional neuronal connections and nerve regeneration. Here we synthesize asymmetric surfaces with well-controlled topography and texture and perform a systematic experimental and theoretical investigation of axonal outgrowth on these substrates. We demonstrate unidirectional axonal bias imparted by the surface ratchet-based topography and quantify the topographical guidance cues that control neuronal growth. We describe the growth cone dynamics using a general stochastic model (Fokker-Planck formalism) and use this model to extract two key dynamical parameters: diffusion (cell motility) coefficient and asymmetric drift coefficient. The drift coefficient is identified with the torque caused by the asymmetric ratchet topography. We relate the observed directional bias in axonal outgrowth to cellular contact guidance behavior, which results in an increase in the cell-surface coupling with increased surface anisotropy. We also demonstrate that the disruption of cytoskeletal dynamics through application of Taxol (stabilizer of microtubules) and Blebbistatin (inhibitor of myosin II activity) greatly reduces the directional bias imparted by these asymmetric surfaces. These results provide new insight into the role played by topographical cues in neuronal growth and could lead to new methods for stimulating neuronal regeneration and the engineering of artificial neuronal tissue.

## Introduction

In the nervous system neuronal cells extend two types of processes, axons and dendrites, which navigate through a complex and dynamic environment to reach their targets and form functional connections. Axonal dynamics is largely controlled by the growth cone, a complex sensory structure located at the leading edge of the axon. Most axons also form branches extending away from the axonal shaft, which is also an important process contributing to the formation of synapses. The growth cone is capable of detecting a large variety of local biochemical, mechanical and topographical cues within the growth environment, and of directing the axonal outgrowth over relatively long distances (hundreds of microns) with remarkable precision [1]–[3]. During this trip, extracellular guidance cues provide critical signals that lead to changes in the motion of the growth cone, including branching, retraction, stops, and turns [3].

Over the past decade, there has been rapid progress in our understanding of the role played by chemical signaling and surface-bound biochemical cues on the growth cone dynamics.

For example, it is known that axonal navigation to their targets depends on the precise spatial arrangement of extracellular matrix proteins on the growth substrates [3]–[5], and that many different types of signal transduction pathways link the activation of growth cone sensors to changes in the internal dynamics of the cytoskeleton [6], [7]. It is also known that surface-bound biochemical cues (*e.g.*, netrins, ephrins, semaphorins) can either attract or repel a growth cone [3], [6], [7]. While chemical guidance cues are relatively well understood, there is recent evidence showing that physical stimuli present in the extracellular environment (external forces, electric fields, as well as substrate physical properties such as stiffness, texture and topography) play a very important role during axonal growth and development [8]–[11]. The growth cone explores its surroundings and guides the axon along a defined path according to spatial landmarks present in the environment.

Much of the insight into the processes that direct axonal elongation in response to mechanical and topographical stimuli comes from *in vitro* studies of neuronal growth on microfabricated substrates. Researchers have used surface patterning to change the physical landscape through which the growth cone navigates and also to direct cellular behavior. For example, it has been shown that altering the substrate stiffness could have dramatic effects on the axonal outgrowth for several types of neurons [9]–[11]. Axonal growth has also been studied on surfaces with different geometrical features such as repeating patterns of symmetric ridges and indentations [12]–[15], parallel lines and gaps or adhesive micro-lines of various geometries [16]–[18], microfluidic channels and 3-dimensional constructs of various sizes [19], [20]. These studies have shown that periodic geometrical features on surfaces increase total axonal outgrowth and tend to bias growth along certain preferred directions, and that the preferred growth orientation with respect to repeating patterns depends on the cell type [15], [21]. Kundu and collaborators have created environments with superimposed topographic and soluble chemical cues, and have demonstrated the existence of an optimal spatial frequency of topographical cues (patterned micropillars), that maximize neurite extension in hippocampal neurons [22]. This work has also studied the combined influence of topographical and chemical cues on neurite extension. However, the mechanisms that control the cellular responses to external mechanical and geometrical stimuli are not completely understood. Furthermore, many of these previous reports provide mostly qualitative descriptions of neuronal growth under the influence of geometrical cues. A quantitative description of the role played by physical stimuli in neuronal outgrowth and of the interactions between these stimuli and growth cones is necessary for a deeper understanding of the fundamental mechanisms that control neuronal growth and the formation of functional synapses. Previous work [23], [24] has shown that the Fokker-Planck equation provides a general framework for predicting the growth cone dynamics and for describing the role that different types of environmental cues have on axonal growth. For example, our group has shown that axonal dynamics for cortical neurons grown on glass substrates coated with poly-D-lysine (PDL) is governed by a V-shaped potential, which results in a regulatory mechanism for the axonal growth rates on these surfaces (24). Another example is provided by the work of Betz and collaborators (23), where the Fokker-Planck formalism was used to quantify the bimodal growth behavior of the leading edge lamellipodia emerging from the internal bistable polymerization processes in the growth cone.

Besides its importance for investigating fundamental growth processes such as growth cone biomechanics and signal transduction mechanisms, the ability to control the neuronal growth on surfaces with controlled geometries and textures has important consequences for neural repair and tissue engineering. Examples include facilitating axonal regeneration following injury, repairing nerve damage, development of artificial neuro-prosthetic interfaces, and creation of implantable devices for nervous system recovery after injury [25], [26]. A critical requirement for all these applications is the ability to direct axonal outgrowth along a single spatial direction (unidirectional bias). While this has been achieved in some cases through electrical stimulation or the application of chemical gradients [27], [28], there are many advantages in using surfaces with well-controlled geometrical properties for regulating neuronal growth. These include absence of electrodes, ability to control short-range signals between cells through spatial confinement, increased re-growth efficiency and tunable response from neurons. A detailed quantitative description of neuronal growth on patterned substrates will therefore enable novel designs of neuronal scaffolds with optimized geometries for implementation in nervous system repair technologies.

Here, we fabricated novel asymmetric surfaces to investigate mechanical and topographical cues for neural guidance and growth. Building on this significant advancement, we quantify axonal dynamics in vitro on substrates with asymmetric topographical cues. Specifically, we use the general theoretical framework based on the Fokker-Planck (F-P) equation to find two key parameters that describe growth cone dynamics: the diffusion (growth cone motility) coefficient and the strength of the effective force imparted by the surface (drift coefficient in F-P equation). We quantify the effects imparted by the surface topography by a single parameter that measures the asymmetry of the surface ratchet. Our results show that the observed asymmetry in the strength of the cell-surface interactions is directly proportional to the asymmetry in the surface ratchet.

## Materials and Methods

### Surface preparation

**Figure 1:**
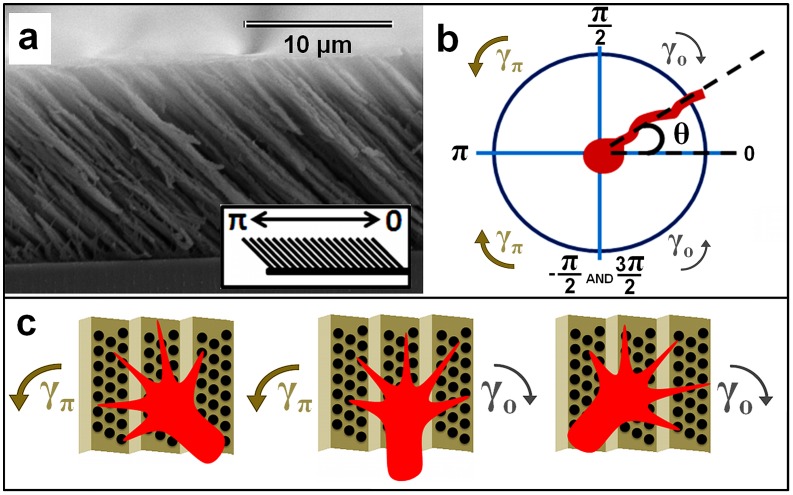
Schematic of growth cone interaction with nano-PPX surfaces. (*a*) SEM image of typical nano-PPX substrate indicating the 0 and *π* radians directions with respect to nanorod tilt. (*b*) Schematic defining the measurement of growth angle *θ* with respect to the *0* and *π* directions. The schematics also show the deterministic torques *γ_o_* and *γ_π_* and the corresponding direction of axonal rotation imparted by these torques. The two angular domains used for data analysis are: −*π/2 ≤ θ ≤ + π/2* and *π/2 ≤ θ ≤ 3π/2*. For the purpose of the analysis −*π/2* is identified with *3π/2* (Figures 3–6 below). (*c*) Schematics of growth cone turning in response to asymmetric torques *γ_π_* and *γ_0_* (growth model described in the main text).

We fabricated asymmetric textured poly(chloro-p-xylylene) (referred to as nano-PPX) surfaces (Fig. 1 *a*) via oblique angle polymerization as described earlier [29]. This surface is hydrophobic and has high hysteresis. Nano-PPX is a flexible material (Young's Modulus ∼ 100 MPa—[30]) and the nanorods can easily deform with shear. We deformed the surface along the nanorod direction by shear using a rubber strip. The shear plastically deforms the surfaces, which are then characterized by AFM.

This preparation resulted in 7 different types of growth substrates, which have been classified based on their topography (see below). The directional surfaces were affixed to glass disks or slides using silicone glue and allowed to dry for a minimum of 48 hours. Once affixed, each surface was rinsed with sterile water, then spin-coated with 3 mL of Poly-D-lysine (PDL) (Sigma-Aldrich, St. Louis, MO) solution (0.1 mg/mL) at 1000 RPM for 10 minutes. The plates were sterilized prior to cell culture using ultraviolet light for ≥30 minutes.

### Cell culture and plating

Isolated rat cortices were obtained from embryonic day 18 rats (Tufts Medical School). The brain tissue protocol was approved by Tufts University Institutional Animal Care Use Committee and complies with the NIH guide for the Care and Use of Laboratory Animals. The cortices were incubated in 5 mL of trypsin at 37°C for 20 minutes. The trypsin was then inhibited with 10 mL of soybean trypsin inhibitor (Life Technologies). The neurons were then mechanically dissociated, centrifuged, the supernatant removed, and the cells were resuspended in 20 mL of neurobasal medium (Life Technologies) supplemented with GlutaMAX, b27 (Life Technologies), and pen/strep. The cells were re-dispersed with a pipette, counted, and plated at a density of 6,000 cells/cm^2^. Each sample was grown for five days prior to measurement.

### Fluorescence microscopy, atomic force microscopy and data analysis

For fluorescence imaging the live cortical samples were rinsed once with phosphate buffered saline (PBS) and then incubated for 30 minutes at 37°C with 50 nM Tubulin Tracker Green (Oregon Green 488 Taxol, bis-Acetate, Life Technologies, Grand Island, NY) in PBS. The samples were then rinsed twice with PBS and immersed in fresh PBS for imaging. Fluorescence images were taken using a standard Fluorescein isothiocyanate -FITC filter: excitation/emission of 495 nm/521 nm. Axon outgrowth was tracked using the NeuronJ plugin for ImageJ (http://rsbweb.nih.gov/ij). For analysis all axons were divided into segments of ∼20 µm per segment. The angle of each segment with respect to the surface direction was measured (see Fig. 1 *b*; nanorods point in the *π* radians direction for all surfaces, as shown in Fig. 1 *a*), and plotted in histograms that quantify angular axonal outgrowth for each type of surface (see below). All surfaces were imaged using an MFP3D Atomic Force Microscope (AFM), using AC mode operation and AC 160TS cantilevers (Asylum Research, Santa Barbara, CA). Surfaces were imaged both before and after neuronal culture, and no significant change in topography was observed.

## Results

### Quantification of the surface topography

The nano-PPX surfaces are composed of tilted nanorods (Fig. 1 *a*) [29], [31]–[33]. The angular distribution of axons on the asymmetric nano-PPX substrates is quantified by the angle *θ*, shown schematically in Fig. 1 *b*: *θ*  =  *π* describes growth in the direction of the tilted nanorods, while *θ*  =  *0* describes growth in the direction opposite to the nanorod tilt. AFM images show that for this type of surface the tilted nanorods clump together to form a ratchet structure, created by oblique angle deposition of PPX nanorods [34]. We will show that this ratchet imparts an effective asymmetric torque on the growth cone (*γ_o_* and *γ_π_* in Fig. 1 *b, c*). Based on the acquired AFM images we introduce a single parameter to characterize each type of surface as described below.

**Figure 2:**
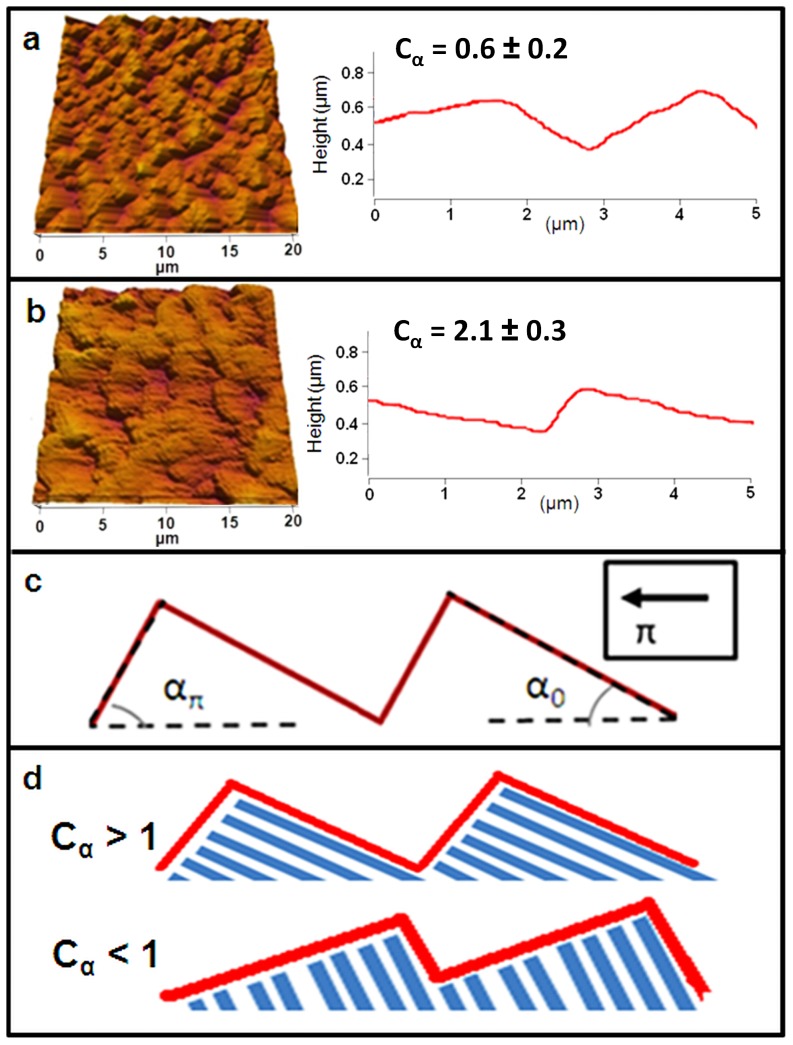
Examples of nano-PPX surface ratchets. (*a*) *Left*: AFM topographical image (20×20 µm) of a nano-PPX substrate where the nanorods point in the *π* radians direction; *Right:* AFM line scan across the substrate illustrating the ratchet topography. The ratchet is oriented in the *0* radians direction (i.e. opposite to the nanorod tilt). (*b*) AFM topographical image (20×20 µm) and AFM line scan illustrating a nano-PPX substrate where the ratchet points in the *π* radians direction (i.e. the same direction as the nanorod tilt). (*c*) Schematics defining ratchet angles *α_0_* and *α_π_* with respect to the *0* and *π* radians directions. d) Schematics illustrating the conditions: *C_α_  =  α_π_/α_0_* > 1 (i.e. nanorods tilt in the same direction as the ratchet structure) and *C_α_  =  α_π_/α_0_* < 1 (i.e. nanorods tilt in the opposite direction to the ratchet structure).

The AFM data (Fig. 2 *a, b* and Fig. S1) shows that the nanorods on each type of surface form a ratchet structure that can be characterized by two angles (shown schematically in Fig. 2 *c, d*): *α_π_* the angle of the side of the ratchet which faces in the nanorod tilt direction (*π* radians in Fig. 2 *c*), and *α_0_*, the angle of the side of the ratchet which faces opposite to the rod tilt direction. Our results show that the ratchet height generally varies between different types of surfaces (see Figure S1) and that both height and slope are important for determining growth properties. To characterize the ratchet asymmetry we define the ratchet angle ratio *C_α_  =  α_π_/α_0_*. We note that for *C_α_>1* the ratchet is oriented in the same direction as the nanorods, and for *C_α_<1* the ratchet is pointing in the direction opposite to the nanorod tilt direction (schematic shown in Fig. 2 *d*). *C_α_ = 1* would indicate a symmetric ratchet. For the unmodified cells in this study we have investigated seven different types of surface ratchets, with a total of 21 substrates distributed as follows: *n = 3* substrates with *C_α_  =  0.6 ± 0.2* (asymmetric ratchets pointing opposite to the rod tilt direction, example shown in Fig. 2 *a*); n =  1 substrates with *C_α_  =  1.26 ± 0.3* (quasi-symmetric ratchet), as well as substrates with *C_α_  =  1.4 ± 0.4 (n = 4), C_α_  =  1.8 ± 0.5 (n = 3), C_α_  =  2.1 ± 0.3 (n = 3), C_α_  =  2.4 ± 0.2 (n = 4)*, and *C_α_  =  3.0 ± 0.9 (n = 3)* (i.e. asymmetric ratchets pointing in the rod tilt direction, examples shown in Fig. 2 *b* and Fig. S1). Experimental uncertainties for *C_α_* are obtained from the standard deviations of measured ratchet angles via AFM. In addition, we have used a total of *n = 14* substrates for the drug-modified cells, as described below.

### Neuronal growth on nano-PPX surfaces

**Figure 3:**
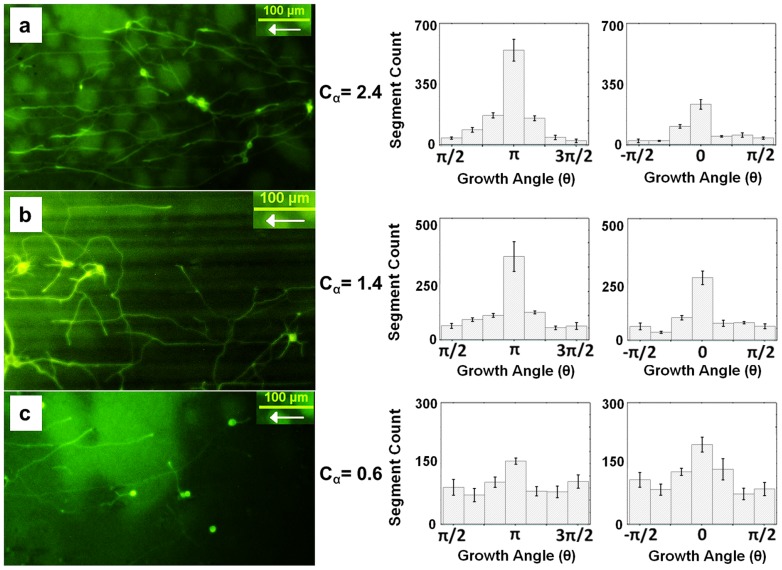
Angular distributions of axonal outgrowth. (*a–c*) Examples of unidirectional axonal outgrowth on nano-PPX substrates with different *C_α_* (as defined in Fig. 2 *c*) and axonal angular distributions both in the nanorod tilt direction (histogram peaks at *π* radians) and opposite to the rod tilt direction (histogram peaks at 0 radians). Axons and cell bodies are shown in green (fluorescence images). Segment count represents the number of axon segments, each one of 20 µm in length. The fluorescent images show representative regions from samples of a given type (labeled by *C_α_*). Axonal growth for unmodified cells was measured on a total number of n = 21 different substrates, distributed as follows: three substrates with *C_α_  =  0.6 ± 0.2*; one substrate with *C_α_  =  1.26 ± 0.3*; four substrates with *C_α_  =  1.4 ± 0.4*; three substrates with *C_α_  =  1.8 ± 0.5*, three substrates with *C_α_  =  2.1 ± 0.3*; four substrates with *C_α_  =  2.4 ± 0.2*, and three substrates with *C_α_  =  3.0 ± 0.9*. The total number of cells measured for each type of substrate was between 160–350. (*a*) Example of growth on nano-PPX surfaces with *C_α_  =  2.4 ± 0.2*. *Left*: representative fluorescence image of neuron outgrowth on this type of surfaces. *Right*: histograms showing the angular distributions of axonal outgrowth centered at *π* and *0* radians, respectively. The histograms show the mean and the standard error of the mean for *n = 4* different substrates with a total of 350 axons (total measured axon outgrowth length on these surfaces is 120 mm). (*b*) *Left*: representative fluorescence image showing axonal outgrowth on a nano-PPX surface with *C_α_  =  1.4 ± 0.4. Right:* histograms showing angular distributions centered at *π* and *0* radians, respectively. The histograms show the mean and the standard error of the mean for *n = 4* different substrates with a total of 312 axons (total measured axon outgrowth length on these surfaces is 106 mm). The maximum outgrowth in (*a–b*) is observed at *π* radians. One way ANOVA followed by pair-wise comparison using Tukey's HSD test shows statistically significant difference between outgrowth centered at *π* vs. *0* radians (p<0.05 for both types of surfaces, see Table S2). (*c*) *Left*: representative fluorescence image showing axonal outgrowth on a nano-PPX surface with *C_α_  =  0.6 ± 0.2. Right:* corresponding angular distributions centered at *π* and *0* radians, respectively. The histograms show the mean and the standard error of the mean for *n = 3* different substrates with a total of 252 axons (total measured axon outgrowth length on these surfaces is 81 mm). The maximum outgrowth in (*c*) is observed at 0 radians (one way ANOVA followed by pair-wise comparison using Tukey's HSD test indicates statistical significance with p<0.05, see table S2). For all angular distributions the maximum outgrowth is always observed in the ratchet direction (*π* radians for *C_α_*>1 and *0* radians for *C_α_*<1). The statistical significance for all types of surfaces is shown by the one-way ANOVA followed by pair-wise comparison using Tukey's HSD test in Table S2 (comparison between pairs of peaks centered at *π* vs. *0* radians, for a given surface type) and Table S3 (comparison between distributions for different values of *C_α_*).

Cortical neurons were grown for five days on each of the seven types of nanorod based ratchet surfaces. Outgrowth was measured by tracing fluorescent images of the axons on each surface. We have previously reported unidirectional growth bias for the un-sheared surfaces, where the tilt angle of the nanorods is 45° [31]. We have demonstrated that axons preferentially extend along the asymmetry of the surface (i.e. direction in which the nanorods are tilted), and display angular distributions that broaden with the increase in the surface density of the cells. In this study we investigate the origin of this asymmetric growth by varying the nanorod tilt angle, and perform a detailed quantitative analysis of the role played by the topographical guidance cues. The data shows that neurons display preferred growth along the surface anisotropy (i.e. in both *θ*  =  *0* and *θ  =  π* directions). Examples of this type of asymmetry for three different values of *C_α_* are shown in the angular distributions presented in Fig. 3 (an additional example for a quasi-symmetric surface with *C_α_  =  1.26 ± 0.3* is shown in Fig. S8).

First, we note that the axonal angular orientation for each type of surface shows clear peaks at both *θ  =  0* and *θ  =  π*. Second, we observe additional *unidirectional* bias for axonal outgrowth for all 6 types of asymmetric surfaces with *C_α_≠1*, i.e. asymmetry in the axonal outgrowth in the *θ = 0* vs. *θ  =  π* direction. This is demonstrated by the statistically significant differences in the peak heights for each pair of angular distributions shown respectively in Fig. 3 *a, b, c*. Statistical significance for comparing the peaks at *π* vs. *0* radians is shown in table S2. Thirdly, the maximum outgrowth is always observed in the direction of the ratchet, for example at *π* radians for *C_α_>1* (Fig. 3, *a* and *b*) and at 0 radians for *C_α_<1* (Fig. 3 *c*), regardless of the underlying direction of the tilted nanorods. Similar results are obtained for all six types of asymmetric surfaces, demonstrating that the observed unidirectional bias is imparted by the underlying surface ratchet topography and does not depend on the nanorod tilt direction. The neurons grown on the quasi-symmetric surface display a significantly reduced unidirectional bias (Fig. S8 and Table S2). As expected, no growth asymmetry or unidirectional bias is observed on control (PDL coated glass) substrates (Fig. S2).

### Theoretical model for neuronal growth

We quantify the influence of ratchet topography on neuronal growth to obtain insight into the growth dynamics on different types of surfaces. In general, the growth cone dynamics can be characterized by two independent parameters, the speed and the growth angle, each variable being described by a general stochastic differential equation (Langevin equation) [23], [24]. We start with the following Langevin equation for the growth angle *θ(t)* that the axon is making with the direction of the surface anisotropy (defined as *0* radians in Fig. 1 *b*):
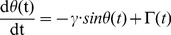
(1)


The magnitude of *γ* quantifies the strength of the effective force imparted by the surface on the growth cone. Γ(*t*) is the “stochastic torque”, which describes the nondeterministic nature of chemotactic and transduction signals (*i.e.,* inter- and intra- cellular signaling). In the absence of this term the model is deterministic and the behavior of a single growth cone could be predicted. We assume a white noise source which has zero mean 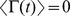 and satisfies the relation: 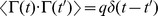, where *q* represents the strength of the noise and *δ* is the Dirac delta function [35].

Equation 1 also describes the motion of human granulocyte cells in the presence of an external electric field [36]. It was shown that human granulocytes move along the direction of the electric field, and the corresponding Langevin equation was derived assuming that cell acts as an automatic controller [36], [37]. A similar equation can be used to characterize the motion of swimming micro-organisms, such as single-cell algae, moving in anisotropic environments (e.g. phototaxis or gravitaxis) [38]. Equation 1 describes the observed growth behavior of axons on nano-PPX surfaces: axons grow preferentially along the direction of the surface anisotropy, and the first term on the right hand side of Eq. 1 has the role of a “deterministic torque”, which tends to rotate the growth cone towards this preferred direction. If the motion of the growth cone is parallel to the direction of surface anisotropy (i.e. *θ = 0* or *θ  =  π*) no cell reaction is necessary, while if the direction of motion is perpendicular to the surface anisotropy (*θ  =  π/2* or *θ  =  3π/2*) the cellular reaction is maximum.

To describe the angular motion of the ensemble of the growth cones one has to solve the corresponding Fokker-Planck equation [35]:
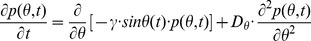
(2)where *p(θ,t)* is the probability distribution for growth angles and *D_θ_* is an effective diffusion (cell motility) coefficient for the *angular* motion.

The angular distributions in Fig. 3 show two types of asymmetry. First, axons display preferred growth in alignment with the surface asymmetry, reflected by the peaks in the angular distributions along the surface anisotropy at *θ  =  π* and *θ = 0* respectively. Second, the histograms in Fig. 3 show polarized axonal growth, i.e. differences in the “left” (*θ*  =  *π*) vs. “right” (*θ* = *0*) motion of the growth cones for each type of surface labeled by a particular value for *C_α_*_._ This polarized growth is reflected by the statistically significant differences in the peak height for each pair of histograms, covering growth in the half-planes: *−π/2 ≤ θ ≤ + π/2* and *π/2 ≤ θ ≤ 3π/2*, respectively. This additional “left-right” asymmetry can be explained by surface curvature - induced effects, leading to asymmetric coupling between the growth cone and the nano-PPX surface ratchet in the “right” vs. “left” directions (see discussion below). To quantify this effect we introduce two different values for the strength of the deterministic torque: *γ_o_* and *γ_π_* (for the “right” and “left” turning, respectively), and write Eq. 2 for the corresponding angular regions: *−π/2 ≤ θ ≤ +π/2* (described by *γ_o_*) and *π/2 ≤ θ ≤ 3π/2* (described by *γ_π_*). The stationary solutions of Eq. 2 for the two regions are:
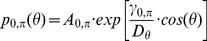
(3)where the indices *0, π* describe the motion in the corresponding angular regions *−π/2 ≤ θ ≤ + π/2* and *π/2 ≤ θ ≤ 3π/2*, respectively (see Fig. 1). *A_0,π_* are two normalization constants given by: 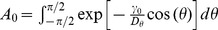 and 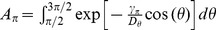.

### Correlations between axonal dynamics and surface topography

**Figure 4:**
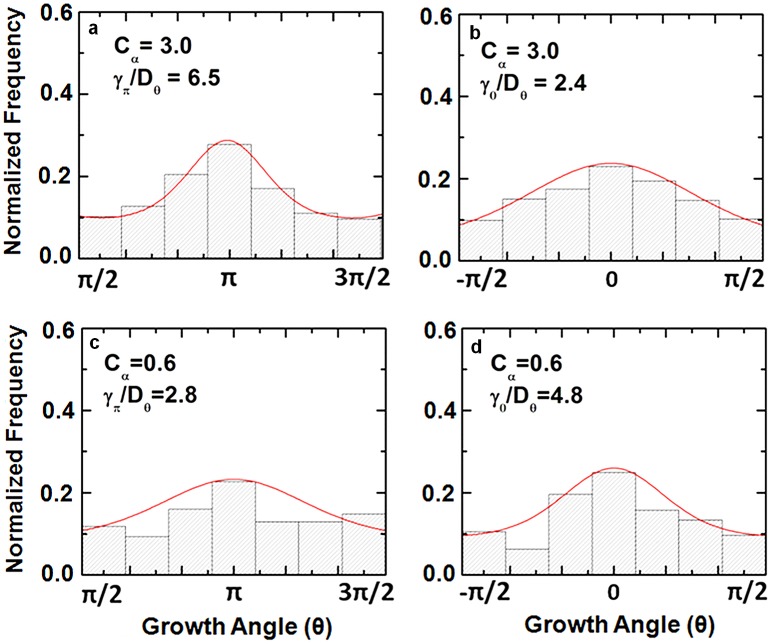
Examples of normalized experimental angular distributions for axonal growth. Normalized experimental angular distributions for axonal growth and fits with Eq. 3 (red curves) for two types of surfaces displaying opposite ratchet asymmetries. (*a*) Normalized angular distribution and fit with Eq. 3 (red curve) for axonal growth in the region *π/2 ≤ θ ≤ 3π/2*, on all substrates with *C_α_  =  3.0 ± 0.9* (i.e. maximum asymmetry in the nanorod direction). (*b*) Normalized angular distribution and fit with Eq. 3 (red curve) for axonal growth in the region *−π/2 ≤ θ ≤ +π/2* on the same type of surfaces as in (a). (*c*) Normalized angular distribution and fit with Eq. 3 (red curve) for axonal growth in the region *π/2 ≤ θ ≤ 3π/2*, on all substrates with *C_α_  =  0.6 ± 0.2* (asymmetric ratchet pointing opposite to the rod tilt direction). (*d*) Normalized angular distribution and fit with Eq. 3 (red curve) for axonal growth in the region *−π/2 ≤ θ ≤ +π/2* on the same type of surfaces as in (*c*). The inset in each figure shows the ratio between the corresponding asymmetric torque (*γ_π_* or *γ_o_*) and the angular diffusion coefficient *D_θ_.* This ratio is obtained from fitting the experimental data with Eq. 3. The total measured axon outgrowth length for (*a*–*b*) is 75 mm (for a total of 241 axons). The total measured axon outgrowth length for (*c*–*d*) is 78 mm (for a total of 273 axons).

Fig. 4 and Fig. S3 show the normalized experimental distributions obtained for all surface types, as well as fits of these distributions with the theoretical model given by Eq. (3).

To determine the effective diffusion coefficient *D_θ_* we perform a best fit for all the experimental angular distributions and select that *D_θ_* which maximizes the likelihood of measuring the given data sets. The FWHM of the peak of the likelihood is used to calculate the error on *D_θ_* (see Fig. S4). We have used a similar procedure for determining the diffusion coefficient of neuron growth cones on glass [24]. With this method a joint fit for all surfaces yields an effective value for the angular diffusion coefficient of 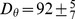
rad^2^/hr.

It is interesting to note that the characteristic speed of growth cones on these surfaces is of the order of *v_c_*∼10 µm/hr, and therefore simple dimensional analysis predicts an effective diffusion coefficient in velocity space of 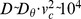 µm^2^/hr^3^. This value for the diffusion coefficient is similar to the value obtained for growth of neuronal cells cultured on glass at similar densities [24]. This result suggest that the stochastic processes involved in the chemotactic and transduction signals are similar for glass and nano-PPX surfaces for cortical neurons cultured at similar surface densities, both types of growth being described by similar effective diffusion coefficients, time scales and characteristic velocities. What is different in the case of the nano-PPX surfaces is the observed uni-directionality of growth, characterized by the parameters *γ_0_* and *γ_π_*. We now turn to the analysis of these parameters for each type of surface topography (ratchet asymmetry) measured experimentally.

**Figure 5:**
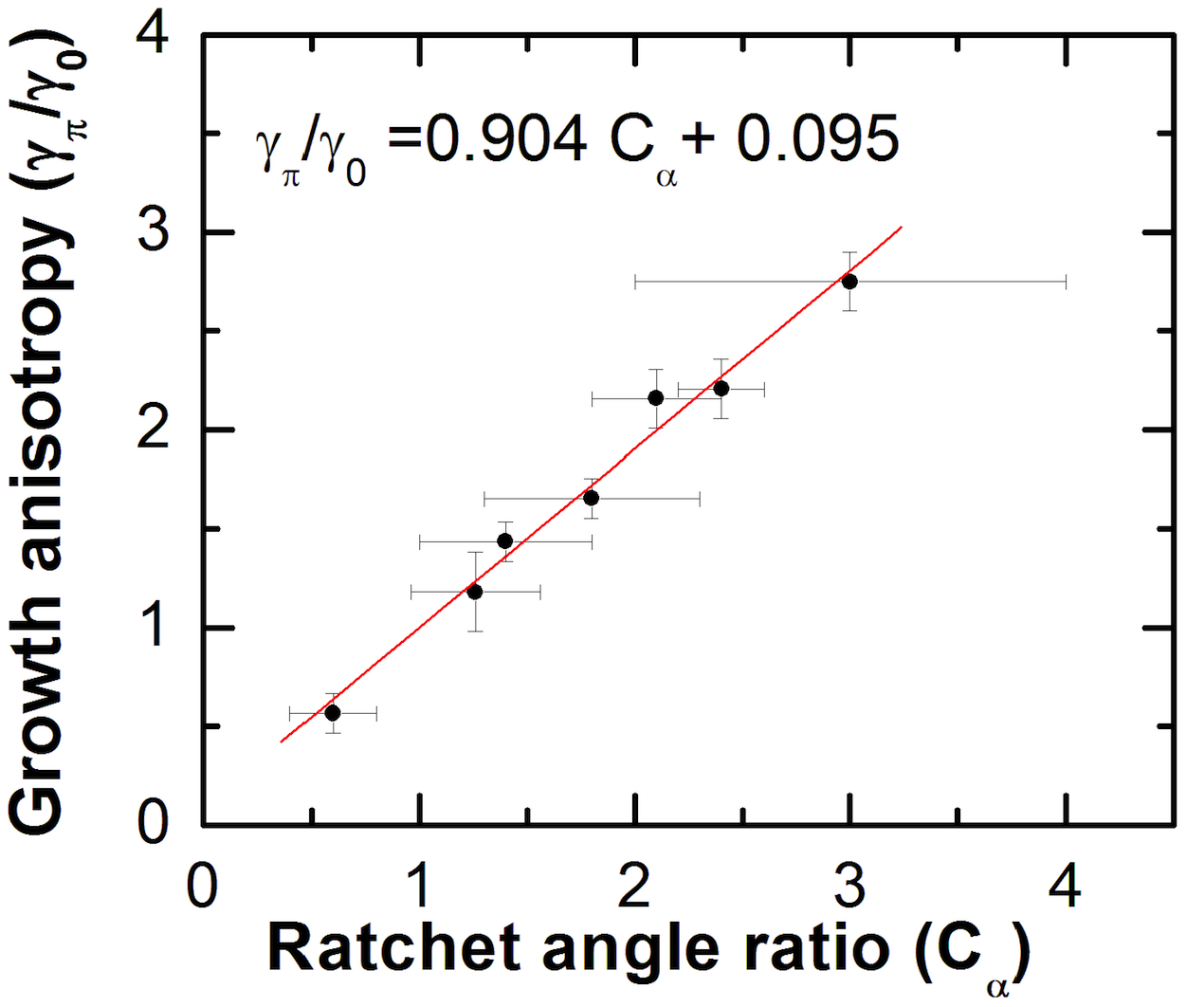
Cell-surface coupling versus ratchet asymmetry. Variation in the strength of the cell-surface coupling asymmetry *γ_π_/γ_0_* with increasing ratchet asymmetry *C_α_* for all 7 types of surfaces measured in the current study. Error bars for the ratchet angle ratios *C_α_* represent experimental uncertainties obtained from the standard deviations of measured ratchet angles via AFM. Error bars for the coupling asymmetry *γ_π_/γ_0_* represent uncertainties obtained from the fit of the normalized angular distributions with Eq. 3.

By fitting the experimental data with Eq. 3 we can quantify the variation of the relative cell-surface coupling torques (given by the ratio *γ_π_/γ_0_* that measures the left-right asymmetry), with ratchet anisotropy (given by the parameter *C_α_*). This dependence is given in Fig. 5, which shows a linear increase in the strength of the cell-surface coupling torque with increasing ratchet asymmetry.

The linear fit of the data in Fig. 5 (red line) crosses within typical outgrowth error of the result (1,1) expected for non-directional or symmetric surfaces (see Fig. S2 and Fig. S8 in the supporting material). We conclude that the asymmetry in the strength of the cell-surface interactions (given by the Fokker-Planck model) is directly proportional to the asymmetry in ratchet angle, measured from AFM topographic images: *γ_π_/γ_0_ ∝ C_α_*. The fact that this relationship is linear, and that the unidirectional bias in axonal growth is always observed in the direction of the ratchet for both *C_α_>1* (nanorods oriented in the direction of the ratchet) and *C_α_<1* (nanorods opposing the ratchet direction) indicates that the direction of nanorod tilt does not contribute significantly to the outgrowth bias. We conclude that the observed growth bias is primarily imparted by the ratchet topography. The ratios between the deterministic torques *γ_π_* or *γ_0_* and the effective angular diffusion coefficient *D_θ_* for each type of surface are given in Table S1 in the supporting material. The two parameters *γ_π_* and *γ_0_* represent the measured bias coefficients in the Fokker-Planck equation, which describes the dynamics of the growth cones on these directional surfaces. The ratio *γ_π_/γ_0_* quantifies the “right” vs. “left” asymmetry in the interactions between cells and surface. The biophysical mechanisms that could give rise to this observed asymmetry are presented in the Discussion section below.

### Effects of Blebbistatin and Taxol on neuronal growth dynamics

To further investigate the influence of topography on the cell-surface coupling we perform two experiments wherein we disrupt the normal functioning of the cytoskeleton and measure the resulting outgrowth on the asymmetric surfaces. We utilize two commonly used cytoskeletal modifying drugs: Taxol (a stabilizer of microtubules) and Blebbistatin (a disrupter of myosin II - mediated actin dynamics) [39], [40].

#### a) Effects of Taxol

**Figure 6:**
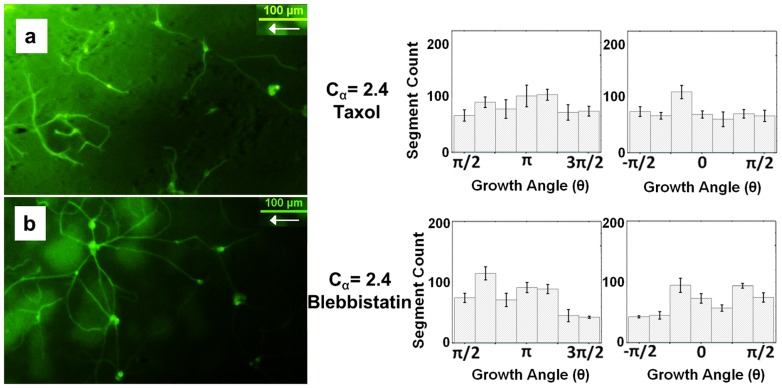
Axon outgrowth for drug-treated neurons on nano-ppx. Examples of axon outgrowth (*left*) and angular distributions for axonal outgrowth (*right*) on nano-PPX surfaces with *C_α_  =  2.4 ± 0.2* for neurons cultured with 10 nM Taxol (*a*) or 10 µM Blebbistatin (*b*). The histograms show the mean and the standard error of the mean for *n = 4* different substrates for each drug. The total measured axon outgrowth length is 68 mm for (*a*) (total of 285 axons) and 91 mm for (*b*) (total of 327 axons). The peaks of the angular distributions at *0* and *π* radians for both (*a*) and (*b*) are clearly reduced compared to the non-treated cells (Fig. 3), indicating a drastic decrease in surface-induced directional growth for drug (Taxol or Blebbisttain) treated cells. One way ANOVA shows no statistically significant difference between outgrowth centered at *π* vs. *0* radians for the drug treated cells (p>0.1, Table S2), demonstrating that there is no unidirectional bias in this case. The ratio between the corresponding asymmetric torque (*γ_π_* or *γ_o_*) and the angular diffusion coefficient *D_θ_* obtained from fitting the experimental data with Eq. 3. is shown in Table S1 and Fig. S5–S7.

To disrupt normal microtubule dynamics we use Taxol (10 nM dose) in the neuronal growth medium at the time of plating. Taxol concentrations higher than 10 nM have been shown to significantly stunt axonal outgrowth [21]. Axonal outgrowth for Taxol modified cells was quantified on two types of surfaces, with *C_α_  =  1.8 ± 0.5* (*n = 2* experimental replicates) and *C_α_  =  2.4 ± 0.2* (*n = 4* experimental replicates), respectively. The Taxol modified cells showed a dramatic decrease in the surface-induced growth directionality (Fig. 6 *a*) compared with the unmodified case (Fig. 3). Statistical significance for comparing growth of taxol-treated vs. non-treated cells is shown in Table S2 and Table S3 in the supporting materials. In addition, fits of the normalized angular distributions for Taxol (Fig. S5) show much smaller values for the deterministic torques *γ_o_* and *γ_π_* (see also Table S1), as well as no unidirectional growth, i.e. *γ_π_ ≈ γ_0_*. While directional axonal outgrowth was greatly reduced by treatment with Taxol, our results show that cells were still growing processes, indicating that growth cone navigation was not inhibited by Taxol.

#### b) Effects of Blebbistatin

To disrupt normal myosin II/actin dynamics in the growth cone we use Blebbistatin (10 µM dose) in the neuronal growth medium at the time of plating. A Blebbistatin concentration of 10 µM has been shown to effectively eliminate traction force in growing Dorsal Root Ganglia neurons [10]. Axonal outgrowth for Blebbistatin modified cells was quantified on two types of nano-PPX surfaces, with *C_α_  =  0.6 ± 0.2* and *C_α_  =  2.4 ± 0.2*, respectively (*n = 4* experimental replicates for each type), for comparison with the unmodified and Taxol modified conditions. Statistical significance for comparing growth of blebbistatin-treated vs. non-treated cells is shown in Tables S2 and S3. Similar to Taxol modification, the Blebbistatin modified cells showed a significant reduction in both directionality in the *0* and *π* radians directions, and left-right coupling asymmetry (see Fig. 6 *b*) as compared with the unmodified cases (see Fig. 3). Fits of the normalized angular distributions for Blebbistatin (Fig. S6) show much smaller values for the deterministic torques *γ_o_* and *γ_π_* (see also Table S1), as well as no unidirectional growth, i.e. *γ_π_ ≈ γ_0_*. Blebbistatin modified cells also showed significant outgrowth, and multiple cell-cell connections, indicating that growth cone navigation was not inhibited by the treatment with Blebbistatin. The Taxol and Blebbistatin experiments demonstrate that microtubules and actin filaments are directly involved in the directional growth, and that the cytoskeleton responds to topographical cues.

## Discussion

The growth cone is a highly dynamic structure that integrates physical stimuli from the growth substrate (*e.g.,* geometrical features, surface texture, substrate stiffness). The growth cone also translates spatial bias into localized cytoskeletal rearrangements that ultimately result in accurate steering and directional growth [1]–[3]. The underlying cytoskeletal mechanisms that drive the growth cone could be affected asymmetrically by external stimuli, and thus introduce spatial bias for steering the growth cone into the right direction according to spatial landmarks [8]–[11]. Moreover, the nervous system has a very heterogeneous and anisotropic architecture, and its mechanical, textural and topographical properties differ considerably between different regions [8]. One thus expects the anisotropy of the growth environment to cause anisotropic migration of the growth cones.

Contact guidance (*i.e.,* the ability of some cells to orient their motion in response to physical structures such as thin lines, bend structures, change in substrate texture etc.) has been observed for many cell types including granulocytes, fibroblasts, and tumor cells. In the case of contact guidance, at every position and orientation the cell obtains the same chemical information but different mechanical and topographical information due to the anisotropy in the substrate physical properties. For example, it was shown that fibroblast (3T3 cells) cultured on pillar substrates display more branched shapes, increased motility and increased surface adhesion compared to similar cells grown on flat surfaces [41]. Neurons have also been reported to grow faster on pillars than they do on flat substrates [42], [43]. In this case, the pillars were considered as “anchoring points” for the growth cone, allowing it to make more rapid progress by reducing the frequency of local searches via protrusion-retraction events. An important parameter for contact guidance is the ratio between the cell size and the characteristic length of the anisotropic features on the surface [11], [37]. This parameter determines the surface density of cell focal adhesion complexes, which mediate adhesion and mechanotransduction between the cell cytoskeleton and the substrate. For example, recent reports have demonstrated that cortical neurons tend to preferentially extend axons in directions perpendicular to the repeating geometrical patterns (microlines and grooves), when the pattern dimension (width and periodicity) is comparable to the size of the growth cone [16]. Furthermore, it was shown that axonal growth on microfabricated pillars is sensitive to the geometry of the micropillar arrangement, and displays maximum response for interpillar spacing of the order of a few microns [22].

Our results demonstrate contact-guidance behavior in the case of cortical neurons cultured on asymmetric nano-PPX surfaces: axons extend preferentially in the direction perpendicular to the periodic ratchet structure. The ratchet periodicity (order of a few microns) is comparable in size with the linear dimension of the cortical neuron growth cone. In addition, growth on nano-PPX surfaces displays unidirectional bias in the sense that the direction of higher motility for growth cones correlates with the ratchet asymmetry given by the parameter *C_α_* (Fig. 5). We suggest that there are at least three possible biophysical mechanisms responsible for the observed growth anisotropy. First, our results suggest a curvature-induced effect for growth cone guidance on these surfaces [11], [41], which tends to maximize topographical guidance as shown schematically in Fig. 1 *c*. Several types of membrane curvature sensing proteins involved in cell adhesion, including amphipathic helices and bin-amphiphysin-rvs (BAR) - domain containing proteins, have been recently identified [11]. Given that the maturation of focal adhesions respond to external forces, including cell-substrate traction forces [44], we hypothesize that the observed “left-right” asymmetry in axonal growth is the result of the differences in left vs. right forces acting on focal adhesions for growth cones advancing along an asymmetric ratchet (Fig. 1 *c*). Second, it has been shown that the directional stimulation of cell motility increases with the density of anchored surface receptors [11], [41]. Thus the focal contacts on a filopodium wrapped over a steep angled feature (high curvature) on the nano-PPX surface will undergo higher forces than those contacting a shallow angled feature, as shown schematically in Fig. 1 *c*. Thirdly, inside the growth cone microtubules act as load-bearing structures, resistant to bending; as such they provide a resistive force to the bending of microtubule-invaded filopodia. Therefore substrate contacts for a filopodium bent around a steeper angle have to resist stronger forces opposing bending, thus exerting larger forces on the integrin-based focal complexes and further inducing the maturation of more focal adhesion points. This positive feedback will result in a larger value of the parameter *γ* on the steeper side of the ratchet. All these effects combined will ultimately lead to higher motility (i.e. larger parameter *γ*) for the growth cones in the direction of larger surface anisotropy, thus providing a biophysical mechanism for the observed directional bias and the “left” vs. “right” anisotropy in the cell-surface coupling.

To our knowledge this is the first study that relates the measured cell growth anisotropy with the effective asymmetry in surface topography (parameter *C_α_*) via a quantitative growth model (Eq. 2 and Fig. 5). We measure a linear relationship between the asymmetry in the cell-surface coupling strength and the ratchet angle ratio: *γ_π_/γ_0_ ∝ C_α_*. This result is consistent with focal contact development increasing with an increase in cell-surface traction forces and with increasing density of anchored surface receptors on the advancing filopodia. This suggests a finely tuned internal mechanism by which the growth cone responds to small changes in topography (curvature and angles of features in the growth environment) rather than to simple detection of external objects.

These conclusions are further supported by the result of our Taxol and Blebbistatin studies. Growth cone motility depends on the dynamic properties of both microtubules and actin filaments. Actin continuously polymerizes and forms actin bundles at the leading edge of the growth cone, and at the same time it is retracted from the leading edge towards the center of the growth cone via a process called F-actin retrograde flow [3]. It has been demonstrated that the F-actin retrograde flow is controlled both by the activity of the motor protein myosin II and by forces exerted by the F-actin bundles present at the periphery (filopodia and lamellipodia) [1], [3]. Focal adhesion complexes create an interface between growth cone and substrate that enable growth cones to exert traction forces on their substrate and to decrease the retrograde flow of F-actin [8]. This leads to actin polymerization at the leading edge and ultimately to the advance (or turning) of the growth cone. Generation of traction forces and directional guidance of the growth cone thus requires myosin II to support neurite tension, control retrograde flow, and mediate forces on the F-actin array. Since Blebbistatin inhibits the activity of myosin II [3], [10], [39], [40] we expect a significant decrease in the growth cone directional bias for neurons treated with this drug. This is indeed the case in our experiments (Fig. 6 *b*), where the Blebbisttain-treated cells display a drastic decrease in surface-induced directional growth (peaks in the angular distribution at *0* or *π* radians), and show no unidirectional bias (asymmetric peaks in the angular histograms at *0* vs. *π* radians due to differences in the “left vs. right” cell-ratchet coupling, Fig. 1 *c*, Table S2, and Table S3). In addition to actin structures, microtubules also have a major role in determining growth cone shape and motility. For example, it has been shown that microtubules in the peripheral domains of growth cone (filopodia and lamellipodia) can act as growth sensors, while microtubules from the bulk central domain can drive the advance of the growth cone [3]. Inhibition of microtubules dynamics via Taxol treatment will therefore prevent growth cone turning in response to topographical cues, as we have indeed observed in our experiments (Fig. 6 *a*).

There are many fundamental stochastic processes that influence growth cone dynamics, including stochastic fluctuations of the very weak (single molecule level) chemical gradients [5], the inherent stochastic nature of biochemical reactions taking place in small, subcellular compartments in the growth cone, dynamic instabilities of the microtubules, bistable polymerization/de-polymerization processes involved in filopodial and lamellipodial dynamics etc. Therefore, quite generally the motion of the growth cone has to be controlled both by a deterministic component (bias to grow in a particular direction determined by chemical gradients, surface topography, stiffness etc.), and a random deviation from this growth direction (i.e. noise in the growth angle) due to stochastic processes. The F-P formalism we have used to analyze the growth on asymmetric surfaces is very general and therefore could be applied to describe growth cone dynamics in response to any type of external cues: topographical, mechanical, biochemical, etc. We also note that there are complex interactions between actin structures and microtubules mediated by associated proteins (dyneins, kinesins, myosins), which can be controlled by asymmetric guidance cues present in the environment [3]. The nano-PPX surfaces could therefore provide a model growth substrate that will enable to investigate how microtubule-actin interactions that steer the growth cone are affected by asymmetric topographical cues.

In conclusion, we have used a stochastic description to analyze growth on asymmetric surfaces, and have extracted key parameters that describe this motion: diffusion coefficient and asymmetric torque imparted by the ratchet topography. To our knowledge this is the first time an asymmetric bias coefficient has been measured for neuronal growth. We have linked the measured anisotropy in axonal outgrowth with the asymmetry in the growth cone-surface coupling due to variations in the ratchet topography. Our findings suggest new strategies for directing neuronal growth in two- and three- dimensional environments, where controlled substrate topography through curvature-induced effects may be used to control the motility of the growth cone and the directionality of axonal outgrowth.

## Supporting Information

Figure S1 **Examples of nano-PPX topographies.** (*a–e*) *Left*: AFM topographical images (20×20 µm) of nano-PPX substrates with different values of *C_α_*. *Right:* AFM line scans across the substrates illustrating ratchet topographies for different values of *C_α_*. Experimental uncertainties for *C_α_* are obtained from the standard deviations of measured ratchet angles via AFM.
(JPG)

Figure S2 **Example of axonal outgrowth on PDL-coated glass (control) surfaces.** *Left:* fluorescence image. *Right*: angular distributions for axon outgrowth on glass surfaces in the regions *π/2 ≤ θ ≤ 3π/2* and *−π/2 ≤ θ ≤ +π/2*, respectively. No growth directionality or asymmetric bias is observed on the glass surfaces. Segment count represents the number of axon segments, each one of 20 µm in length. Error bars represent standard error of the mean over n = 4 different samples. The total measured axon outgrowth length on this surface is 107 mm (for a total number of 294 axons).
(JPG)

Figure S3 **Normalized experimental angular distributions and fits with Eq. 3 (red curves) for axonal growth on surfaces with different topographies, given by different values for** ***C_α_*** **.** The histograms on the left column display normalized angular distributions in the region: *π/2 ≤ θ ≤ 3π/2*. The histograms on the right column display normalized angular distributions in the region: *−π/2 ≤ θ ≤ +π/2*. The inset in each figure shows the ratio between the corresponding asymmetric torque (*γ_π_* or *γ_o_*) and the angular diffusion coefficient *D_θ_*. This ratio is obtained from fitting the experimental data with Eq. 3. The total measured axon outgrowth length varies between 69 mm (total of 256 axons for surfaces with *C_α_  =  2.1 ± 0.3*) and 115 mm (total of 327 axons for surfaces with *C_α_  =  1.8 ± 0.5*).
(JPG)

Figure S4
**Maximum likelihood fitting for** ***D_θ_*** **.** For *N* measured angles *θ_i_*, each with associated probability distribution *p(θ)*, the likelihood function is defined as: 
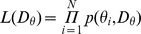
. The value of the diffusion coefficient *D_θ_* that maximizes *L* is the best-fit value. The reported error on this value is the FWHM of the peak in the likelihood function. Here, the natural log of the likelihood function is presented for all data combined, and the ln(2) is subtracted from the maximum to find the FWHM (blue line). A similar procedure was used to find the diffusion coefficient of growth cones on glass and is described in reference [24].(JPG)

Figure S5 **Normalized experimental angular distributions and fits with Eq. 3 (red curves) for axonal growth for neurons treated with Taxol (10 nM) on surfaces with** ***C_α_  =  2.4 ± 0.2*** **.** The inset in each figure shows the ratio between the corresponding asymmetric torque (*γ_π_* or *γ_o_*) and the angular diffusion coefficient *D_θ_*. No unidirectional bias is observed in this case, (*γ_π_* ≈ *γ_o_*), indicating no difference in the “left vs. right” cell-ratchet coupling (see main text).
(JPG)

Figure S6 **Normalized experimental angular distributions and fits with Eq. 3 (red curves) for axonal growth for neurons treated with Blebbisttain (10 µM) on surfaces with** ***C_α_  =  2.4 ± 0.2*** **.** The inset in each figure shows the ratio between the corresponding asymmetric torque (*γ_π_* or *γ_o_*) and the angular diffusion coefficient *D_θ_*. No unidirectional bias is observed in this case (*γ_π_* ≈ *γ_o_*), indicating no difference in the “left vs. right” cell-ratchet coupling (see main text).
(JPG)

Figure S7 **Example of axonal outgrowth on nano-PPX surfaces with** ***C_α_  =  0.6 ± 0.2*** **, for neurons treated with 10 µM of Blebbistatin.** *Left:* fluorescence image of axonal outgrowth. *Right*: angular distributions for axon outgrowth on these surfaces in the regions *π/2 ≤ θ ≤ 3π/2* and *−π/2 ≤ θ ≤ +π/2*, respectively. The peaks of the angular distributions at *0* and *π* radians are clearly reduced compared to the non-treated cells. This is similar to the case of treated cells grown on surfaces with *C_α_>1* (Fig. 6). Segment count represents the number of axon segments, each one of 20 µm in length. Error bars represent standard error of the mean over n = 4 different substrates. The total measured axon outgrowth length on this surface is 22 mm (for a total of 128 axons).
(JPG)

Figure S8 **(a) Examples of axonal outgrowth on a quasi-symmetric substrate with** ***C_α_  =  1.26 ± 0.3*** **, and axonal angular distributions both in the nanorod tilt direction (histogram peaks at π radians) and opposite to the rod tilt direction (histogram peaks at 0 radians).** Axons and cell bodies are shown in green (fluorescence images). Segment count represents the number of axon segments, each one of 20 µm in length. Error bars represent standard error of the mean over 4 different data sets collected on the same substrate. The total measured axon outgrowth length on this surface is 57 mm (167 axons in total) A significant reduction in the unidirectional bias is observed in this case (p value for one way ANOVA is given in Table S2; values for *γ_o_* and *γ_π_* are given in Table S1).
(JPG)

Table S1 Summary of the ratios between the deterministic torques *γ_π_* (and *γ_0_* respectively) and the effective angular diffusion coefficient *D_θ_* for each type of surface labeled by *C_α_*. Experimental uncertainties for *C_α_* are obtained from the standard deviations of measured ratchet angles via AFM. The ratios between the deterministic torques and the angular diffusion coefficient are obtained from the fit of the normalized angular distributions (Fig. 4 and Fig. S3) with the theoretical model given by Eq. 3. The quoted uncertainties in these ratios are the standard errors obtained for the best-fit parameters (95% confidence interval).
(JPG)

Table S2 Summary of p values for one-way ANOVA followed by pair-wise comparison using Tukey's HSD test, comparing the peaks centered at *π* vs. *0* radians for all surface types. The small values (p<0.05) obtained for non-treated cells on all asymmetric surfaces indicate statistically significant differences between axonal outgrowth in the two directions. Cells grown on a quasi-symmetric surface (*C_α_*  =  1.26 ± 0.3) show significantly reduced difference between the peaks. Cell treated with Blebbistatin and Taxol do not show a statistically significant difference between the two peaks (p>0.1).
(JPG)

Table S3 **Examples of comparing angular distributions between different pairs of surfaces.** The table shows the summary of p values for one-way ANOVA followed by pair-wise comparison using Tukey's HSD test for the types of surfaces shown in Fig. 3 and Fig. 6. Only the average values for *C_α_* are shown in the first column. The small p values (p<0.05) indicate statistically significant differences between axonal growth on different pairs of surfaces, and between the growth of non-treated vs. treated cells.
(JPG)
