# Supplementary figures and images for: Effects of Surface Asymmetry on Neuronal Growth
Source: PLoS One. 2014 Sep 3;9(9):e106709. doi: 10.1371/journal.pone.0106709 (PMC4153665; doi:10.1371/journal.pone.0106709)

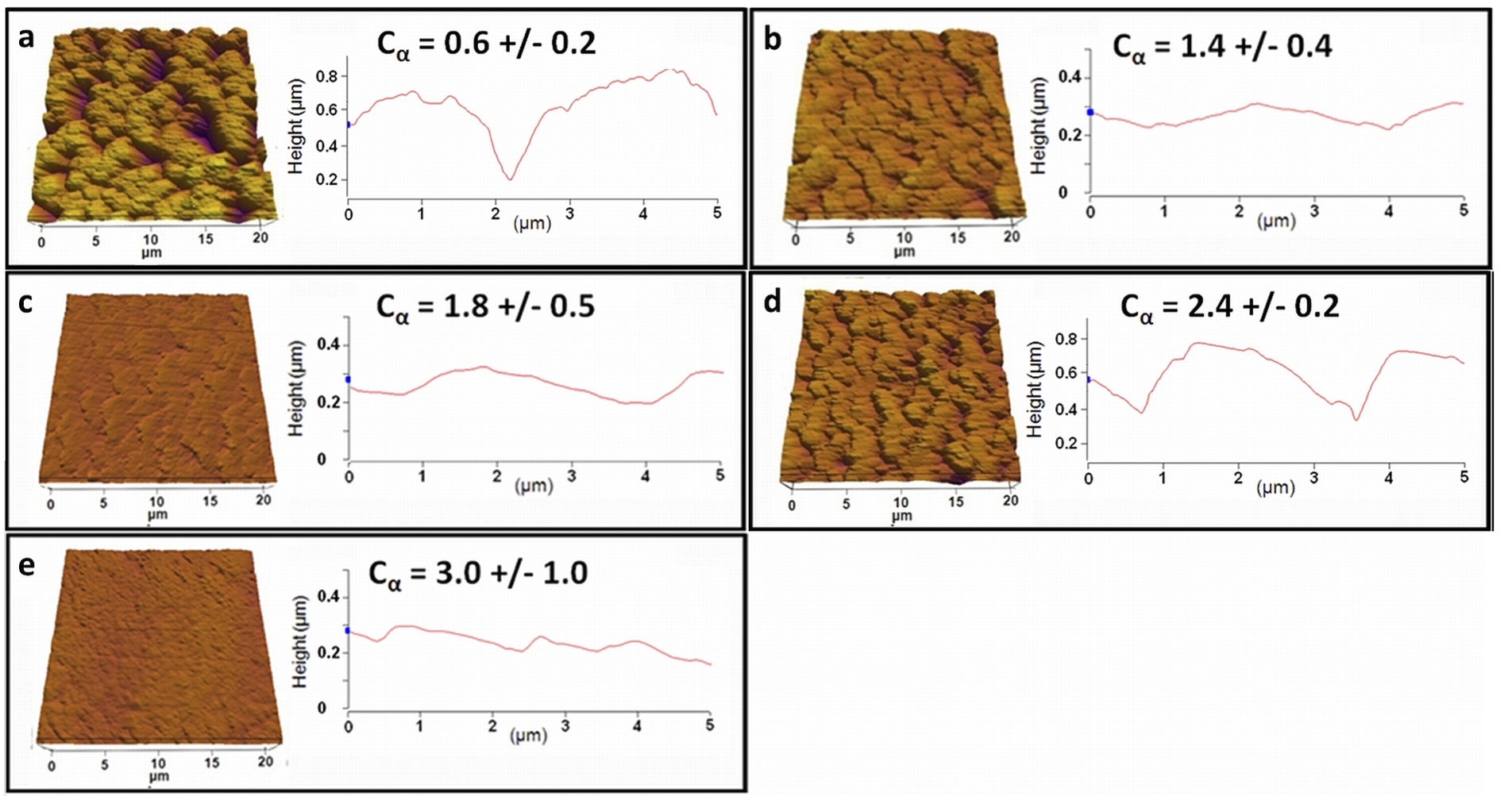

Supplement: Figure S1 — Examples of nano-PPX topographies. (a–e) Left: AFM topographical images (20×20 µm) of nano-PPX substrates with different values of Cα. Right: AFM line scans across the substrates illustrating ratchet topographies for different values of Cα. Experimental uncertainties for Cα are obtained from the standard deviations of measured ratchet angles via AFM. (JPG) [file pone.0106709.s001.jpg]

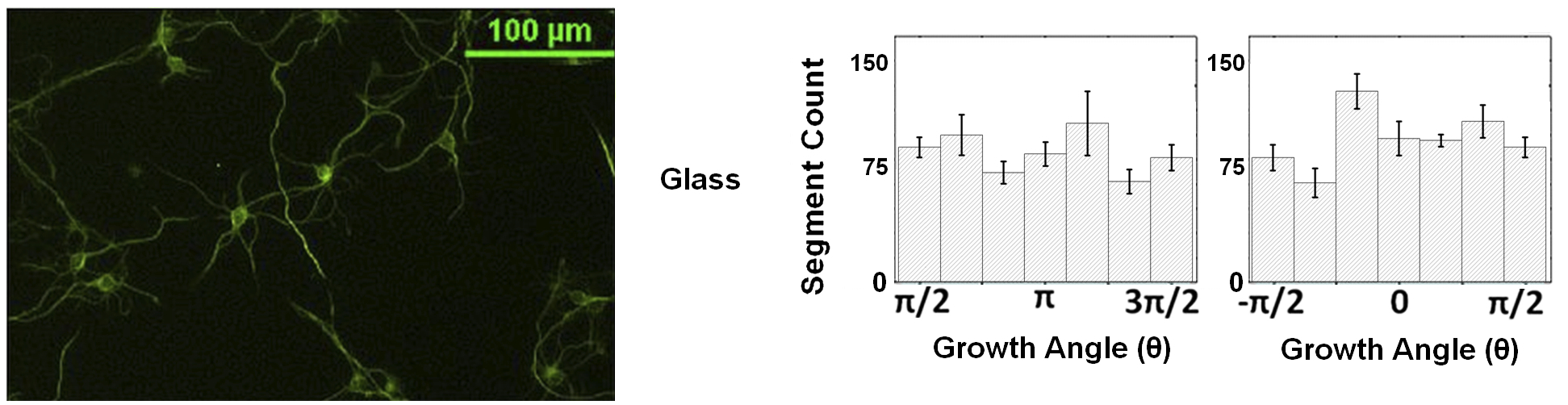

Supplement: Figure S2 — Example of axonal outgrowth on PDL-coated glass (control) surfaces. Left: fluorescence image. Right: angular distributions for axon outgrowth on glass surfaces in the regions π/2 ≤ θ ≤ 3π/2 and −π/2 ≤ θ ≤ +π/2, respectively. No growth directionality or asymmetric bias is observed on the glass surfaces. Segment count represents the number of axon segments, each one of 20 µm in length. Error bars represent standard error of the mean over n = 4 different samples. The total measured axon outgrowth length on this surface is 107 mm (for a total number of 294 axons). (JPG) [file pone.0106709.s002.jpg]

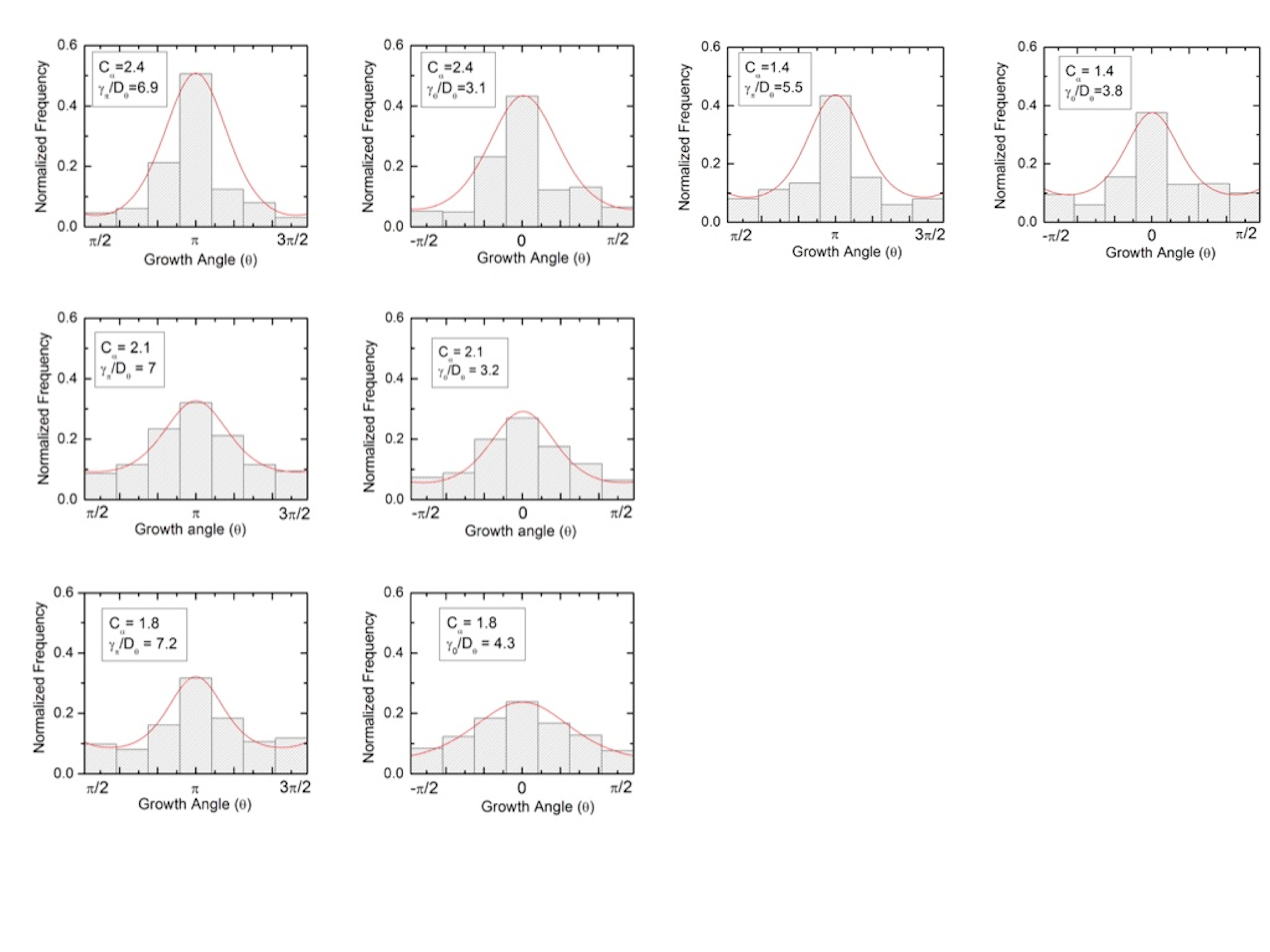

Supplement: Figure S3 — Normalized experimental angular distributions and fits with Eq. 3 (red curves) for axonal growth on surfaces with different topographies, given by different values for Cα . The histograms on the left column display normalized angular distributions in the region: π/2 ≤ θ ≤ 3π/2. The histograms on the right column display normalized angular distributions in the region: −π/2 ≤ θ ≤ +π/2. The inset in each figure shows the ratio between the corresponding asymmetric torque (γπ or γo) and the angular diffusion coefficient Dθ. This ratio is obtained from fitting the experimental data with Eq. 3. The total measured axon outgrowth length varies between 69 mm (total of 256 axons for surfaces with Cα = 2.1 ± 0.3) and 115 mm (total of 327 axons for surfaces with Cα = 1.8 ± 0.5). (JPG) [file pone.0106709.s003.jpg]

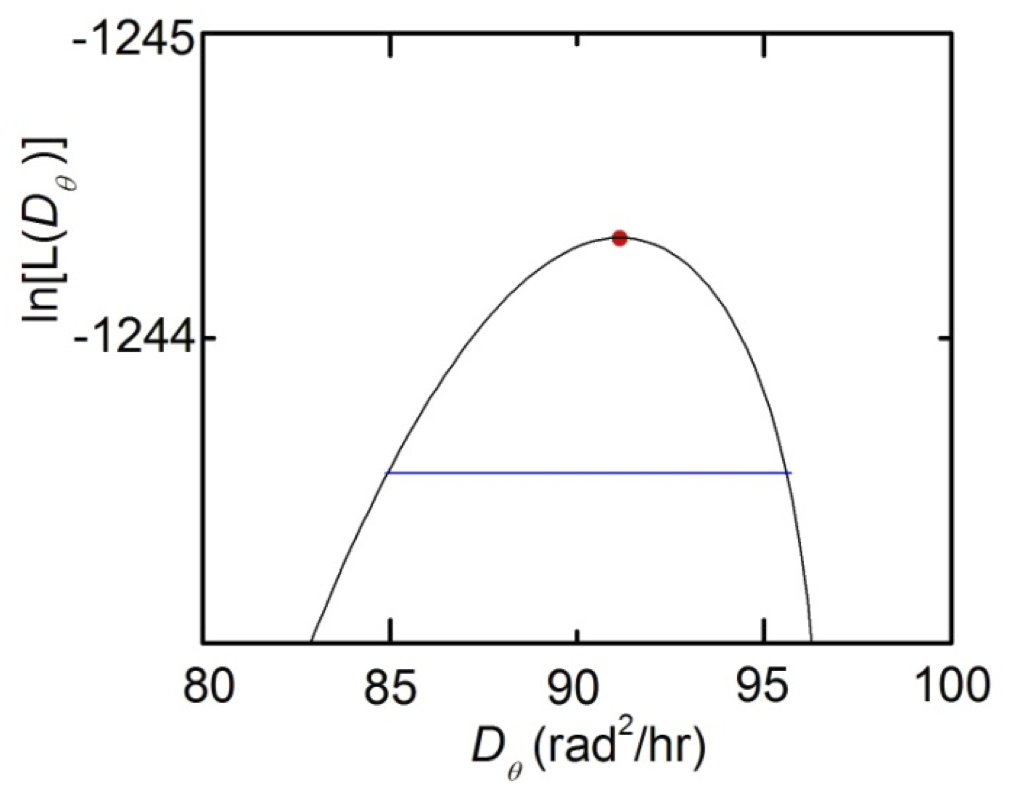

Supplement: Figure S4 — Maximum likelihood fitting for Dθ . For N measured angles θi, each with associated probability distribution p(θ), the likelihood function is defined as: . The value of the diffusion coefficient Dθ that maximizes L is the best-fit value. The reported error on this value is the FWHM of the peak in the likelihood function. Here, the natural log of the likelihood function is presented for all data combined, and the ln(2) is subtracted from the maximum to find the FWHM (blue line). A similar procedure was used to find the diffusion coefficient of growth cones on glass and is described in reference [24]. (JPG) [file pone.0106709.s004.jpg]

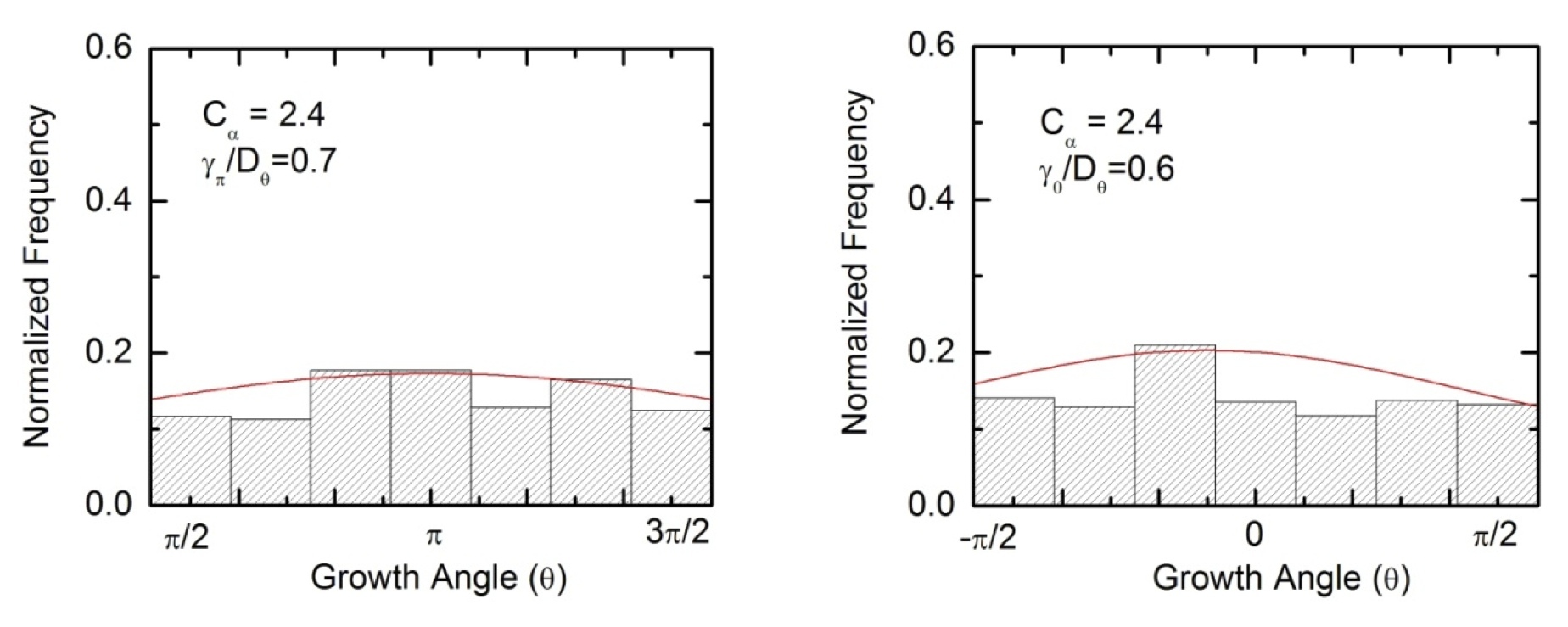

Supplement: Figure S5 — Normalized experimental angular distributions and fits with Eq. 3 (red curves) for axonal growth for neurons treated with Taxol (10 nM) on surfaces with Cα = 2.4 ± 0.2 . The inset in each figure shows the ratio between the corresponding asymmetric torque (γπ or γo) and the angular diffusion coefficient Dθ. No unidirectional bias is observed in this case, (γπ ≈ γo), indicating no difference in the “left vs. right” cell-ratchet coupling (see main text). (JPG) [file pone.0106709.s005.jpg]

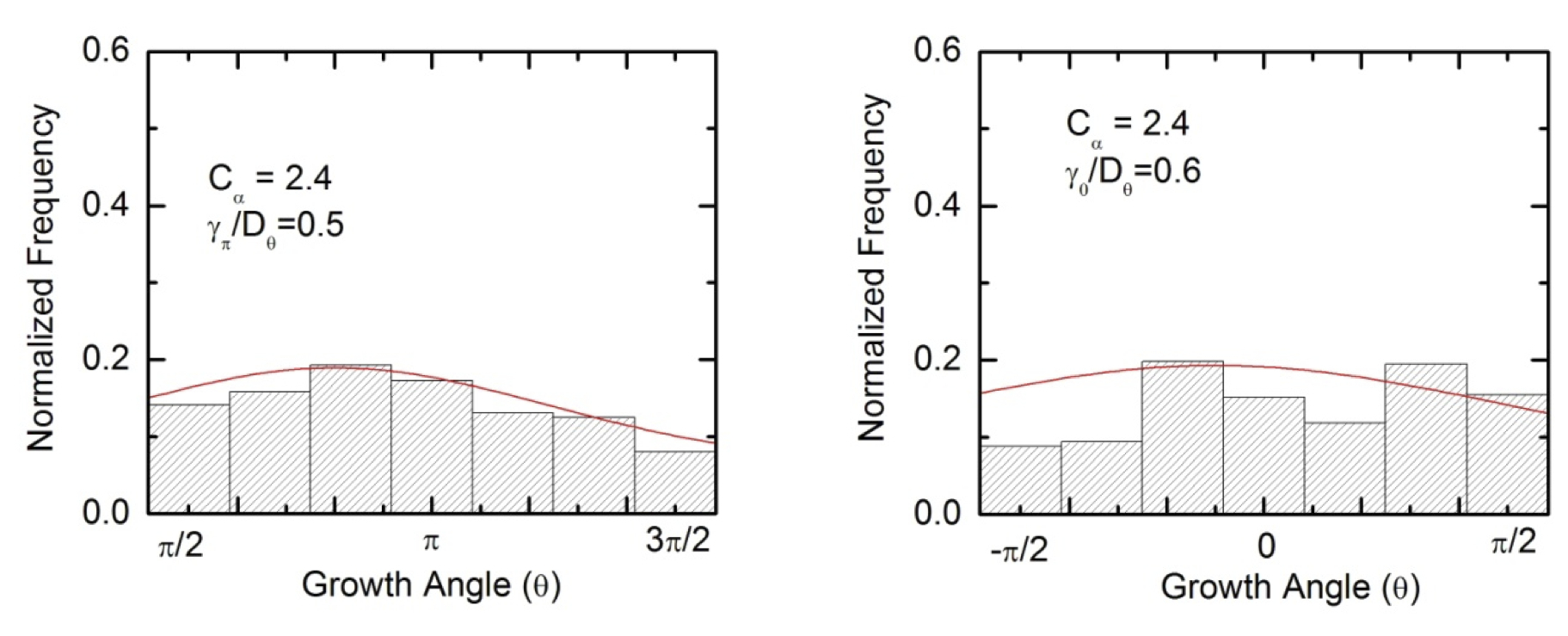

Supplement: Figure S6 — Normalized experimental angular distributions and fits with Eq. 3 (red curves) for axonal growth for neurons treated with Blebbisttain (10 µM) on surfaces with Cα = 2.4 ± 0.2 . The inset in each figure shows the ratio between the corresponding asymmetric torque (γπ or γo) and the angular diffusion coefficient Dθ. No unidirectional bias is observed in this case (γπ ≈ γo), indicating no difference in the “left vs. right” cell-ratchet coupling (see main text). (JPG) [file pone.0106709.s006.jpg]

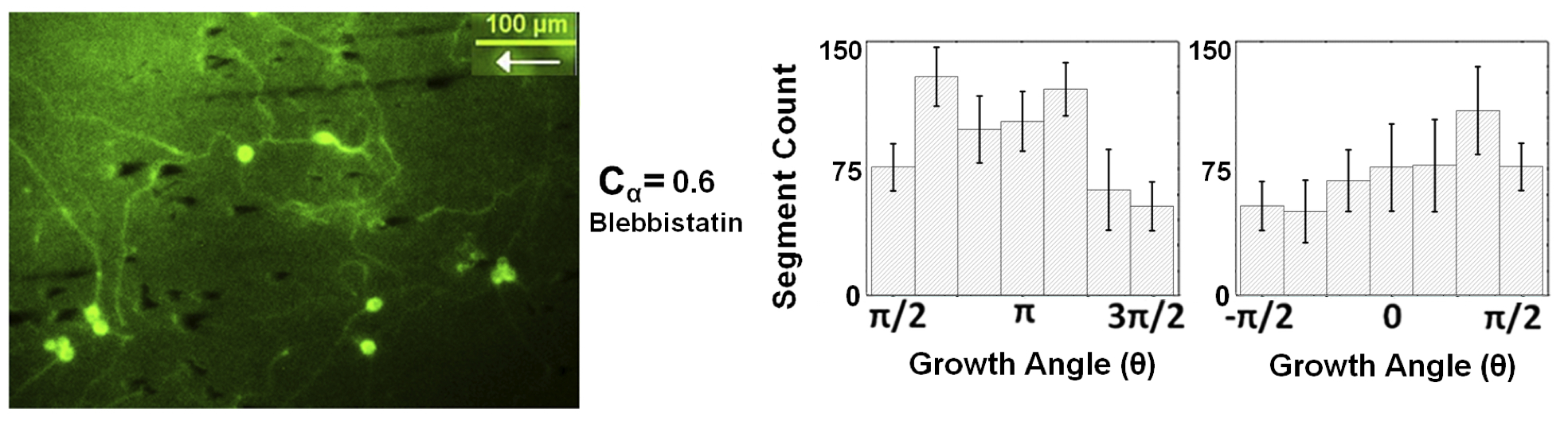

Supplement: Figure S7 — Example of axonal outgrowth on nano-PPX surfaces with Cα = 0.6 ± 0.2 , for neurons treated with 10 µM of Blebbistatin. Left: fluorescence image of axonal outgrowth. Right: angular distributions for axon outgrowth on these surfaces in the regions π/2 ≤ θ ≤ 3π/2 and −π/2 ≤ θ ≤ +π/2, respectively. The peaks of the angular distributions at 0 and π radians are clearly reduced compared to the non-treated cells. This is similar to the case of treated cells grown on surfaces with Cα>1 (Fig. 6). Segment count represents the number of axon segments, each one of 20 µm in length. Error bars represent standard error of the mean over n = 4 different substrates. The total measured axon outgrowth length on this surface is 22 mm (for a total of 128 axons). (JPG) [file pone.0106709.s007.jpg]

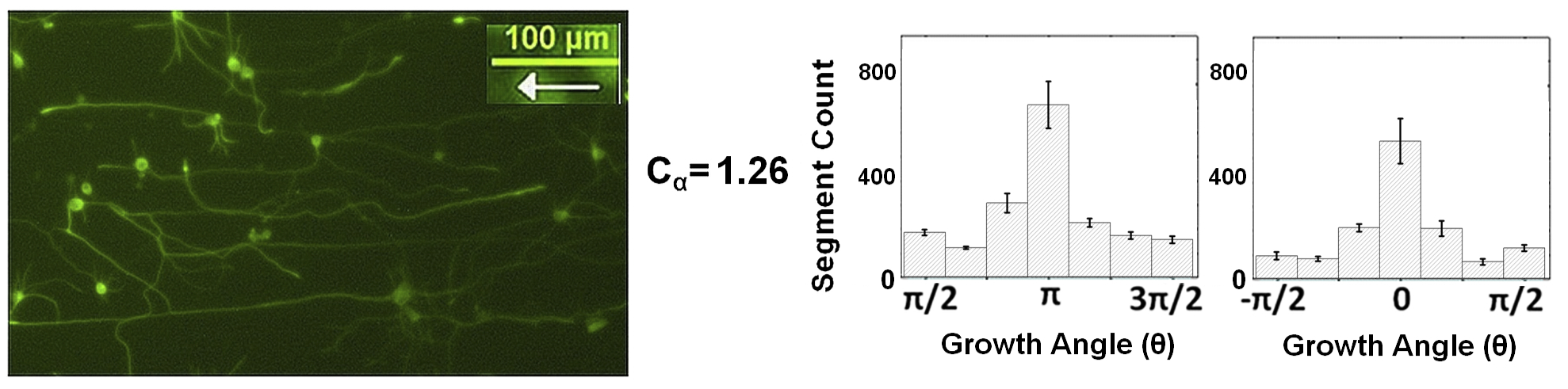

Supplement: Figure S8 — (a) Examples of axonal outgrowth on a quasi-symmetric substrate with Cα = 1.26 ± 0.3 , and axonal angular distributions both in the nanorod tilt direction (histogram peaks at π radians) and opposite to the rod tilt direction (histogram peaks at 0 radians). Axons and cell bodies are shown in green (fluorescence images). Segment count represents the number of axon segments, each one of 20 µm in length. Error bars represent standard error of the mean over 4 different data sets collected on the same substrate. The total measured axon outgrowth length on this surface is 57 mm (167 axons in total) A significant reduction in the unidirectional bias is observed in this case (p value for one way ANOVA is given in Table S2; values for γo and γπ are given in Table S1). (JPG) [file pone.0106709.s008.jpg]

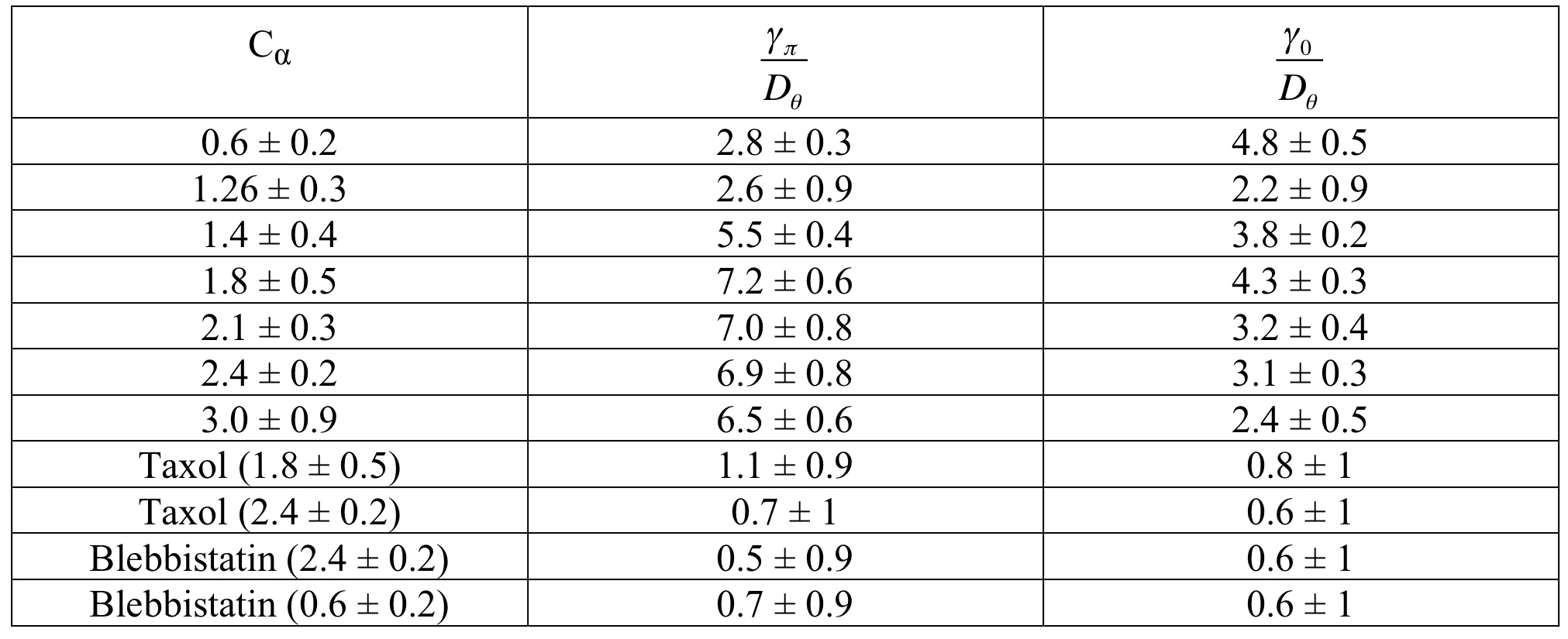

Supplement: Table S1 — Summary of the ratios between the deterministic torques γπ (and γ0 respectively) and the effective angular diffusion coefficient Dθ for each type of surface labeled by Cα. Experimental uncertainties for Cα are obtained from the standard deviations of measured ratchet angles via AFM. The ratios between the deterministic torques and the angular diffusion coefficient are obtained from the fit of the normalized angular distributions (Fig. 4 and Fig. S3) with the theoretical model given by Eq. 3. The quoted uncertainties in these ratios are the standard errors obtained for the best-fit parameters (95% confidence interval). (JPG) [file pone.0106709.s009.jpg]

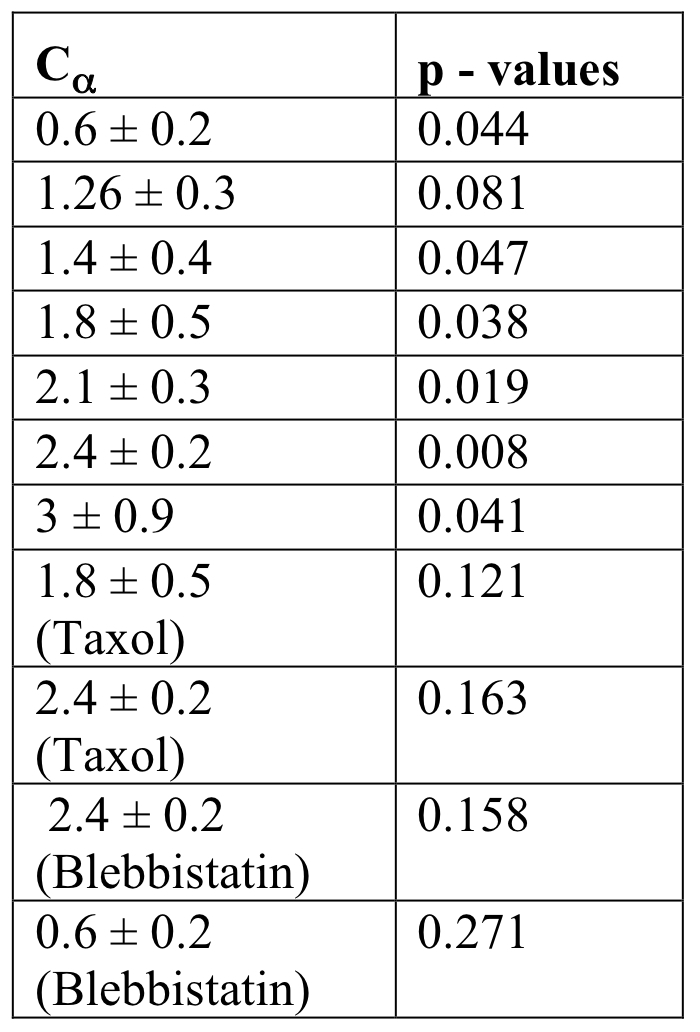

Supplement: Table S2 — Summary of p values for one-way ANOVA followed by pair-wise comparison using Tukey's HSD test, comparing the peaks centered at π vs. 0 radians for all surface types. The small values (p<0.05) obtained for non-treated cells on all asymmetric surfaces indicate statistically significant differences between axonal outgrowth in the two directions. Cells grown on a quasi-symmetric surface (Cα = 1.26 ± 0.3) show significantly reduced difference between the peaks. Cell treated with Blebbistatin and Taxol do not show a statistically significant difference between the two peaks (p>0.1). (JPG) [file pone.0106709.s010.jpg]

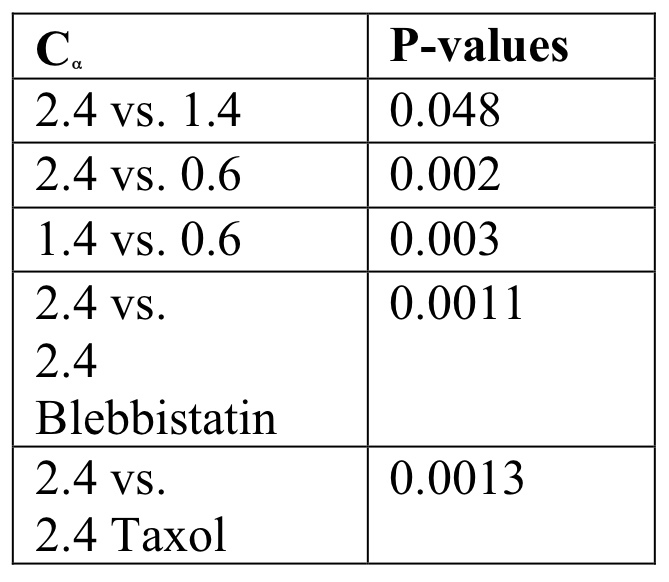

Supplement: Table S3 — Examples of comparing angular distributions between different pairs of surfaces. The table shows the summary of p values for one-way ANOVA followed by pair-wise comparison using Tukey's HSD test for the types of surfaces shown in Fig. 3 and Fig. 6. Only the average values for Cα are shown in the first column. The small p values (p<0.05) indicate statistically significant differences between axonal growth on different pairs of surfaces, and between the growth of non-treated vs. treated cells. (JPG) [file pone.0106709.s011.jpg]
